# The New South London Hospital for Women

**Published:** 1912-10-19

**Authors:** 


### THE NEW SOUTH LONDON HOSPITAL FOR WOMEN.

Whenever any proposal to found a new hospital in London is mooted, it must be very carefully scrutinised as to principles and details by experts, because it appeals for funds to a public which cannot be expected to be conversant with the soundness or otherwise of the enterprise. Lady Eleanor Cecil in giving details of the proposed Women's Hospital bases her appeal mainly on the grounds of the necessities of the female portion of the working-class population of South London and of the Southern Counties. She claims that working women hesitate about consulting male practitioners concerning their maladies, whether minor ailments or serious diseases, and that the demands upon the Royal Free Hospital and the Euston Road Hospital are greater than can be met. With this plea we are very considerably in sympathy. Speaking generally, the needs of the hospital population in London are adequately met by the beds available in the various voluntary hospitals; indeed, we believe that they are more than met at the present moment. (We are leaving out of account the possible effects of the Insurance Act, since the contingencies that may thus arise cannot at present be accurately estimated.) If there is an exception to this broad rule it is in respect of the diseases peculiar to women. We should not like to say that there is any actual shortage of beds allotted to gynæcological cases, but the margin in hand is small, and the pressure on the accommodation available is greater. Hence we may grant that if a new special hospital is required, one for women is the most useful that can be proposed. Further, the south side of the Thames is less well served in this respect than the north, though it must not be thought that we under-value in the least the splendid services of Guy's and St. Thomas's or the Royal Waterloo Hospital. Further still, it is undeniable that some women prefer to consult members of their own sex rather than a male practitioner; nor is it at all surprising that they should do so.

At the same time we believe that many women prefer male attendance, and that the prevalence of the desire for women doctors is in danger of being over-estimated. It is undeniable that the marvellous advances in gynæcological surgery of recent years have been entirely the work of male workers, notwithstanding the fact that the Royal Free and Euston Road Hospitals have been established all through the Listerian epoch, during the latter half of which the greatest strides have been made. It may be mere masculine prejudice, but we do not honestly think the best women gynæcologists in London are up to the standard of efficiency attained by the best men in the same specialty. Perhaps when the new hospital has been running for a few years we may alter this opinion.

Turning from broad principles to the details of the scheme, we note that a site for an in-patient department facing Clapham Common has been secured, and that part of the projected hospital will consist of paying wards at fees of one to three guineas a week. This proposal seems to us admirable. Clapham is a district which—formerly inhabited by the very affluent—now contains a large population of those who cannot afford to pay West End nursing-home charges, and yet are better educated and better off than the usual hospital class. The neighbouring regions of Brixton, Battersea, and Wandsworth also contain a large working-class population, as well as many for whom these paying wards will be a great boon. At present the Bolingbroke is the only local hospital serving this large and densely populated region. Lady Eleanor Cecil does not disclose whether practitioners not on the staff of the hospital will be allowed to treat their own patients in these wards; nor—if so—do we learn whether women practitioners only will enjoy this privilege. An out-patient department will be opened shortly, it is also announced, near the termini of the great southern railways, and will thus be easily accessible. The purchase of the site and the erection and equipment of the hospital are estimated to cost at least £25,000, of which £6,000 has already been received by the committee. We await with interest the further development of the scheme.

